# Results of Acellular Dermis Matrix graft used for Tympanoplasty in Guinea pig model

**Published:** 2015-03

**Authors:** Farhad Farahani, Alireza Karimi Yazdi, Mohammad Ghasemi, Elnaz Shariatpanahi, Abdol-Mohammad Kajbafzadeh, Saeid Amanpour

**Affiliations:** 1 *Otolaryngology Research Center, Tehran University of Medical Sciences, Tehran, Iran. *; 2 *Sinus and Surgical Endoscopic Research Center, Ghaem Hospital, School of Medicine, Mashhad University of Medical Sciences, Mashhad, Iran.*; 3 *Department of Otolaryngology, School of Medicine, Hamadan University of Medical Sciences & Health Services, Hamadan, Iran.*; 4 *Pediatric Urology Research Center, Children’s Hospital Medical Center, Tehran University of Medical Science, Tehran, Iran.*; 5 *Cancer Model Research Center, Cancer Institute of Iran, Tehran University of Medical Sciences, Tehran, Iran.*

**Keywords:** Partial perforation, Total perforation, Tympanoplasty.

## Abstract

**Introduction:**

To describe the underlay tympanoplasty technique using an acellular dermal graft(AlloDerm) for tympanic membrane (TM) reconstruction in a guinea pig model and to demonstrate the feasibility of the technique using AlloDerm tissue harvested from the prepuce as a source of tissue for future grafting in human TM reconstruction.

**Materials and Methods:**

The prepuce was divided during circumcision and the acellular dermis was prepared using a number of standard processes. Two groups of guinea pigs were prepared. In the case group (20 guinea pigs and 40 ears) removal of TM was performed with tympanoplasty using AlloDerm, and in the control group (eight guinea pigs and 16 ears), removal of TM was performed without tympanoplasty. In each group, the TM was completely removed in one ear and partially removed on the other side, and the integrity of the TMs was re-evaluated after 8 weeks.

**Results:**

In the case group, the healing rates in the completely and partially removed TMs were 83.3% and 94.4%, respectively. The difference in healing rate (0% and 66.7%, respectively) was statistically significant (P<0.05).

**Conclusion:**

The use of AlloDerm is safe and effective in the repair of TM perforations in a guinea pig model. Acceptable results of AlloDerm tympanoplasty in a guinea pig model may pave the way for the effective use of this material in human TM reconstructions.

## Introduction

Tympanic membrane (TM) perforations are a common otologic problem (1). Temporalis fascia, perichondrium, cartilage–perichondrium composite grafts , pressed scar tissue grafts, poly (glycerol sebacate)-engineered plugs, and water-soluble chitosan patches are all materials used for the repair of TM perforations (2-5). The most commonly used source of graft material for TM repair is the temporalis fascia and its overlying loose areolar tissue. The ease of harvest through a standard postauricular incision, high success rate, and established safety has popularized these tissues for TM grafting. Problems may be encountered when a revision tympanoplasty procedure is required, as in the case of recurrent disease or a planned second-look procedure, because sufficient fascia may no longer be readily available. In such a situation, a surgeon may be forced to find other tissue sites for grafting (6). A variety of autograft, allograft, xenograft, and alloplast materials have been used in the surgical closure of a TM perforation (7). TM repair has also been described with the use of acellular connective tissue materials (8). 

Tissue engineering is an emerging multidisciplinary field in which researchers are striving to replace or regenerate damaged or lost tissues. The principle common to tissue engineering is to produce new tissues by seeding cells onto a suitable three-dimensional scaffold in an appropriate growth environment. According to this principle, AlloDerm can be regarded as a natural acellular scaffold to facilitate TM healing (4). It is derived from de-epithelized acellular human cadaver dermis and can be used successfully in many fields of surgery (9). AlloDerm not only acts as an allogenic substitute, but also provides a dermal skeleton to which peripheral skin cells can migrate (10). Several studies have demonstrated the efficacy of AlloDerm in closing small TM perforations (11). Fishman et al. evaluated the use of AlloDerm for total TM reconstruction primarily in adults, and demonstrated a high closure rate and a significantly shortened healing time (12). Because of legal and ethical considerations, it is desirable to demonstrate the safety and effectiveness of this technique in an animal model before using AlloDerm in human tympanoplasty.

The objective of this study was to demonstrate the feasibility of using AlloDerm tissue harvested from the prepuce as a source of tissue for future grafting in human TM reconstruction through use of a guinea pig model.

## Materials and Methods

This experimental study was performed in the Imam Khomeini hospital complex of Tehran University of Medical Sciences with the cooperation of the Pediatric Urology Research Center.

AlloDerm was supplied by the above center, and prepuce was selected as an available, cheap and easily transportable source of AlloDerm preparation. The prepuce was divided during circumcision under sterile conditions and was maintained in Roswell Park Memorial Institute (RPMI) solution containing penicillin and streptomycin at 4 °C. Then the prepuce (on the ice) was transferred under a laminar hood and its mucus was divided, before being placed on a dish containing saline solution and put into the incubator at 38 °C for 48 hours. After this time, the prepuce was transferred under the laminar hood again for de-epidermization, and its epidermis was collected and picked up gently. Then, the de-epidermization solution was aspirated, and 20 ml of Hanex solution was added to the bottle before the bottle was rotated three times at 60 revolutions per minute (rpm) for 5 minutes. In the next stage, the prepuce was added to 1% sodium dodecyl sulfate (SDS) solution for 90 minutes and was transferred to the rotator revolving at 60 rpm. The SDS solution was substituted every 30 minutes. Then the prepuce was put into Hanex solution on a rotator revolving at 60 rpm for 20 minutes thrice, and then added to 1% Trigon solution X-100 for 20 minutes. After this stage, the prepuce was processed with 0.5% trypsin and 2% ethylenediaminetetraacetic acid (EDTA) at 37 °C for 30 minutes. Then the acellular prepuce was washed in phosphate buffered saline (PBS) solution for 30 minutes thrice and was maintained in the PBS solution containing penicillin and streptomycin until cell culturing.

In this study, 28 guinea pigs were divided into a case and a control group (the sample size in each group was determined by statistical analysis; and in order to respect animal research ethical codes, the minimal sample size for the control group was selected). Twenty guinea pigs were selected as a case group. The integrity of the TMs and middle ears of these guinea pigs were confirmed by inspection under a microscope and then using tympanometry in the audiology service. Then under general anesthesia (intramuscular injection of 10 mg/kg ketamine and 50 mg/kg xylocaine) and under sterile conditions and with the aid of a microscope and routine microsurgery instruments for the ear, the TM of one side of each guinea pig was completely removed with the tympanic ring (total perforation) and underlay tympanoplasty was performed with a cellular dermis and without trauma to the ossicular chain, with endaural incision. On the other site, the TM was partially removed (partial perforations) in anteroinferior and posteroinferior quadrants (the tympanic ring was maintained) and tympanoplasty was performed in the same manner as previously described. After surgery, 40 mg/kg trimethoprim and 20 mg/ml sulfadiazine were intramuscularly injected for prophylaxis of infection. The integrity of the TMs of both sides was evaluated by otomicroscopy and tympanometry 8 weeks after surgery and the success rate of tympanoplasty was assessed.

Eight guinea pigs were selected as the control group. Removal of TM was performed in the manner described for the case group, but tympanoplasty was not performed and the TMs were left for possible spontaneous healing (no other material such as fascia for tympanoplasty was used in this group to evaluate the role of AlloDerm in the success rate of tympanoplasty when compared with spontaneous healing). Also in the control group, for evaluation of possible undesirable immunologic reactions, a piece of AlloDerm was implanted subdermally in the forearm of each guinea pig. The amount of AlloDerm was similar to that required for bilateral tympanoplasty (to ensure approximately similar antigenic load with the case group). Again, both ears of the guinea pigs were evaluated 8 weeks after surgery in the same manner as the case group, and subdermally implanted AlloDerms were removed and sent to the pathology service for evaluation of inflammatory changes.

After data collection, all statistical analysis was performed using SPSS software (version 15.0, SPSS Inc., Chicago, Illinois). Statistical values were determined using the Fisher exact test. P≥0.05 was considered to be statistically significant. 

## Results

From 28 guinea pigs, four (two in each case and control group) were deceased by the end of the study. Eighteen guinea pigs remained in the case group. Among the ears with complete TM removal (total perforations), three grafts (16.7%) failed and 15 grafts (83.3%) healed. In the other ear of the guinea pigs in this group (partially removed TMs or partial perforations), one graft (5.6%) failed and 17 grafts (94.4%) healed. In the control group, six out of eight guinea pigs survived. Among the ears with complete TM removal (total perforations), none (0%) healed spontaneously. In the other ear (partially removed TMs or partial perforations), four TMs (66.7%) healed spontaneously and intact TMs were confirmed in these cases. In two cases (33.3%), TMs had a perforation (Table 1). Rate of healing of TM in the case group (tympanoplasty with AlloDerm) was statistically significant (P<0.05) in comparison with the control group (damage to the TM without tympanoplasty).

Pathologic assessment of subdermally implanted AlloDerm in the control group indicated slight chronic and nonspecific inflammatory changes without necrosis or foreign-body reactions.

**Table 1:** Results of tympanic membrane healing with and without tympanoplasty in two groups of guinea pigs

**Group**		**Healed**		**Failed**		**Total**
		**number**	**Percent**	**number**	**Percent**	
Case	Completely removal	15	83.3%	3	16.7%	18
	Partially removal	17	94.4%	1	5.6%	18
	Total	32	88.9%	4	11.1%	36
Control	Completely removal	0	0%	6	100%	6
	Partially removal	4	66.7%	2	33.3%	6
	Total	4	33.3%	8	66.7%	12

## Discussion

Temporalis fascia remains the most commonly used material for TM reconstruction. It has a very high success rate and offers several advantages (8). Revision tympanoplasty and second-look procedures are common in otologic surgeries (13). Cholesteatoma may persist or recur despite clinically apparent complete surgical removal. Additionally, Eustachian tube dysfunction frequently persists, resulting in re-retraction or perforation. Previous temporalis fascia harvest may not leave sufficient tissue for a second graft at revision surgery. At this point, the surgeon must find another source of graft material. Traditionally, this material may include allografts from a second surgical site such as the contralateral temporalis fascia or another tissue such as the auricular and septal perichondrium, cartilage, vein, or pericranium (1). TM repair has also been described with the use of AlloDerm (acellular dermis) (14).

AlloDerm is an acellular dermal graft that is processed from banked cadaver skin. The cellular elements are removed in the processing of the allograft but the native collagen and elastin matrix and the basement membrane complex (BMC) are preserved. It is also treated with agents that prevent any viral transmission when implanted. Because the tissue is acellular, it does not produce an antigenic inflammatory response after implantation (7). Originally described as a permanent dermal allograft in wound grafting, AlloDerm has been reported in facial soft tissue augmentation, intraoral resurfacing, and repair of nasal septal perforations (15-22).

Several studies illustrate the use of AlloDerm in tympanoplasty. Fayad et al. studied 24 patients undergoing tympanoplasty for chronic otitis media. Successful perforation closure was achieved in 87.5% of cases (23). A study by Lai et al. demonstrated that the use of AlloDerm achieved comparable rates of successful TM repair and hearing improvement with fascia in children using both medial and lateral grafting techniques (24). Vos et al. found comparable rates of TM healing with AlloDerm. In addition, a statistical reduction in surgical time was noted with use of an AlloDerm graft verses both fascia and cartilage tympanoplasty (25).

In this study we used AlloDerm for tympanoplasty in guinea pigs. Because of legal and ethical considerations, it is desirable to demonstrate safety and effectiveness of this technique in animal models before it is used in human tympanoplasty. According to similar animal model studies in otologic surgeries we followed-up the cases for 8 weeks and demonstrated that use of AlloDerm is safe and effective in the repair of TM perforations in a guinea pig model. Furthermore, we did not see any adverse inflammatory reaction against AlloDerm. In general, this technique was successful in guinea pig models, and for better evaluation we recommend comparison of the results of AlloDerm tympanoplasty with other available materials such as the fascia, vein, and perichondrium in future animal studies. 

## Conclusion

The use of AlloDerm is safe and effective in the repair of TM perforations in a guinea pig model. Acceptable results of AlloDerm tympanoplasty in a guinea pig model may pave the way for the effective use of this material in human TM reconstructions.
